# Identify Beta-Hairpin Motifs with Quadratic Discriminant Algorithm Based on the Chemical Shifts

**DOI:** 10.1371/journal.pone.0139280

**Published:** 2015-09-30

**Authors:** Feng YongE, Kou GaoShan

**Affiliations:** College of Science, Inner Mongolia Agriculture University, Hohhot, PR China; University of Michigan, UNITED STATES

## Abstract

Successful prediction of the beta-hairpin motif will be helpful for understanding the of the fold recognition. Some algorithms have been proposed for the prediction of beta-hairpin motifs. However, the parameters used by these methods were primarily based on the amino acid sequences. Here, we proposed a novel model for predicting beta-hairpin structure based on the chemical shift. Firstly, we analyzed the statistical distribution of chemical shifts of six nuclei in not beta-hairpin and beta-hairpin motifs. Secondly, we used these chemical shifts as features combined with three algorithms to predict beta-hairpin structure. Finally, we achieved the best prediction, namely sensitivity of 92%, the specificity of 94% with 0.85 of Mathew’s correlation coefficient using quadratic discriminant analysis algorithm, which is clearly superior to the same method for the prediction of beta-hairpin structure from 20 amino acid compositions in the three-fold cross-validation. Our finding showed that the chemical shift is an effective parameter for beta-hairpin prediction, suggesting the quadratic discriminant analysis is a powerful algorithm for the prediction of beta-hairpin.

## Introduction

Protein function is inherently correlated with its structure. So, the prediction of protein structure is an active research field in bioinformatics. At present, it is still difficult to predict the spatial structure directly from protein primary structure. However, the successful prediction of protein super-secondary structure is the key step in the spatial structure prediction. Protein super-secondary-structure motifs are composed of a few regular secondary structural elements connected by loops. These structural motifs play an important role in protein folding and stability because a large number of motifs exist in protein spatial structure. Generally speaking, the empirical prediction of protein super-secondary structure essentially consists of two parts: one is the prediction of different structural types from amino acid sequences [1–3]; another is the prediction of structural motifs [4–7]. In this article we concentrate on the latter. The prediction of beta-hairpin motif will be helpful to identify fold in the unknown structure. In the past decade, many researchers have focused on exploring methods for beta-hairpin prediction [6–10]. However, the features of these studies were mainly derived from the amino acid compositions or dipeptide compositions. In this study, we introduced a novel feature, chemical shifts (CSs), to predict beta-hairpin motifs. Chemical shift describes the local chemical environment of nuclear spins in nuclear magnetic resonance [11]. Therefore, some researchers have utilized it for the determination of bimolecular structures and molecular dynamics studies [12–17]. Moreover, some works have studied on protein structure prediction [18–26] and protein backbone and side chain torsion angle prediction [27] by using chemical shifts, results showing that chemical shift is a powerful parameter for the determination of protein structure information.

In this paper, we would like to utilize CSs as parameters to predict beta-hairpin motifs combined with quadratic discriminant analysis. Using the benchmark dataset, we adopted three-fold cross-validation and achieved the sensitivity of 92% and specificity of 94% and the overall prediction accuracy of 87% by using CSs of six nuclei as features and combining with quadratic discriminant analysis (QDA) algorithm. At the same time, to compare with other parameter, we have performed the prediction by using 20 amino acid compositions (AAC) as inputs of the method of QDA. The results showed that the performance of CSs outperform that of 20 AAC in the prediction of beta-hairpin. At present, some machine learning algorithms were used in the prediction of beta-hairpin motifs [6–10]. Therefore, to test our method and facilitate comparison with other methods, we have performed the prediction by using the same six CSs as feature of the support vector machine (SVM) and Random forest (RF) algorithm in the same cross-validation. Compared results showed that QDA is better than the other two algorithms in terms of accuracies.

## Materials and Methods

### Database

All of the CSs data used in this paper were retrieved from the re-referenced protein chemical shift database RefDB [28]. The following steps were performed to construct our dataset. Firstly, only proteins in RefDB overlapping with the corresponding Protein Data Bank (PDB) file with sequence identity of 100% were considered. Secondly, only proteins with the beta-hairpin or beta-link (called not beta-hairpin) motifs information in ArchDB40 database [29] were considered. Thirdly, only proteins with six nuclei (*C*,*C*_*α*_,*C*_*β*_,*H*_*N*_,*H*_*α*_,*N*) assigned CSs were considered. Finally, we utilized the PISCES program [30] to remove the highly similarity sequences. After strictly following the aforementioned procedures, 123 proteins were obtained. Among 123 proteins, 87% (107 sequences) proteins have less than 25% sequence identity, and the sequence identity of the remains ranges from 25 to 30%. In 123 proteins, due to consider the six CSs information at the same time, finally we obtained 157 beta-hairpin fragments, in which the lengths are ranged from 7 to 38 amino acid residues. And 75 not beta-hairpin fragments, the lengths of these fragments are ranged from 8 to 40 amino acid residues. PDB IDs of 123 and CSs data of 157 beta-hairpin fragments and 75 not beta-hairpin fragments are listed in the Supplementary Materials S1–S3 files.

### Feature parameter

In the two data subsets {beta-hairpin, not beta-hairpin}, we calculated the averaged CSs of six nuclei for a fragment of length *l* using following formula.

$$t_{m}=\frac{1}{l}\sum_{j=1}^{l}CS_{m}^{j}$$ (1)

Here $l=\left\{\begin{array}{l} {}[7\sim 38]\;in\;beta-hairpin\;dataset\\ {}[8\sim 40]\;in\;not\;beta-hairpin\;dataset \end{array}\right\}$,*m* = *C*,*C*_*α*_,*C*_*β*_,*H*_*N*_,*H*_*α*_,*N*,and *j* represents amino acid positions in the fragment. Therefore, a sequence fragment can be converted into a six-dimensional vector *R*:{*t*_*m*_}.

### Statistical distribution

Under the normal distribution, the analysis of variance (ANOVA) can be used to test whether there was a significant difference for two-group or multi-group samples [19, 31] in the database. In this paper, the ANOVA is defined by Eq (2)

$$MS_{T}=MS_{B}+MS_{W}$$ (2)

where *MS*_*T*_, *MS*_*B*_ and *MS*_*W*_ denoted the square means of total, between groups and within a group, respectively. The statistical value, called *F*-value, is the ratio of *MS*_*B*_ and *MS*_*W*_, which can be calculated by Eq (3)

$$F\text{-value}=MS_{B}/MS_{W}$$ (3)

From Eq (3), we can see that the *MS*_*B*_ becomes increasingly larger than *MS*_*W*_, *F*-value will become larger. That is to say, there are significant differences between groups, otherwise, the lack of differences.

### Quadratic discriminant analysis (QDA)

As mentioned above [6–10], various parameters such as amino acid compositions and dipeptide compositions have been employed in the prediction of beta-hairpin. Here, we used CSs as feature to predict beta-hairpin motifs.

The QDA [32–35] is an effective algorithm that has been widely applied in genomic and proteomic bioinformatics in recent years. Thus, we used it here to perform prediction.

For a sequence *X* to be classified, we calculated the averaged CSs of six nuclei using the Eq (1). So, the sequence is converted into a six-dimensional vector *R*:{*t*_*m*_}

$$R:\{t_{m}\}\;(m=C,C_{\alpha },C_{\beta },H_{N},H_{\alpha },N)$$ (4)

Here we integrated six-dimensional vector by using QDA. Consider a sequence *X* is classified into two groups (beta-hairpin, not beta-hairpin). The discriminant analysis function between group *i* and group *j* is defined by

$$\xi_{ij}=\text{ln}p(\omega_{i}|X)-\text{ln}p(\omega_{j}|X)$$ (5)

According to Bayes’ Theorem, we deduce

$$\begin{array}{l} \xi_{ij}=\text{ln}\frac{p_{i}}{p_{j}}-\frac{\delta_{i}-\delta_{j}}{2}-\frac{1}{2}\text{ln}\frac{\left|\Sigma_{i}\right|}{\left|\Sigma_{j}\right|}\\ \;=(\text{ln}p_{i}-\frac{1}{2}\delta_{i}-\frac{1}{2}\text{ln}\left|\sum_{i}\right|)-(\text{ln}p_{j}-\frac{1}{2}\delta_{j}-\frac{1}{2}\text{ln}\left|\sum_{j}\right|) \end{array}$$ (6)

Set

$$\eta_{v}=\text{ln}p_{v}-\frac{\delta_{v}}{2}-\frac{1}{2}\text{ln}\left|\sum_{v}\right|$$ (7)

where

$$\delta_{v}={\left(R-\mu_{v}\right)}^{T}\;\sum_{v}^{-1}\;(R-\mu_{v})$$ (8)

where *v* = beta-hairpin, not beta-hairpin, and *p*_*v*_ denotes the number of samples in group *v*, δ_*v*_ is the square mahalanobis distance between *R* and μ_*v*_ with respect to Σ*v* (notes: *μ*_*v*_ and |Σ_v_| are calculated in training set), and *μ*_*v*_ denotes chemical shift values of six nuclei *R*:{*t*_*m*_} averaged over group *v*, |Σ_v_| is the determinant of matrix Σ_v_.

The six-dimensional vector *μ*_*v*_ can be written

$$\mu_{m}^{\left(v\right)}=\frac{1}{p_{v}}\sum_{n=1}^{p_{v}}t_{m}^{n}\;$$ (9)

here *p*_*v*_ denotes the number of samples in group *v*; $t_{m}^{n}$ tdenotes the average CSs of *m* nuclei for *n*-th sequence in group *v*; *v* = beta-hairpin, not beta-hairpin; *m = C*,*C*_*α*_,*C*_*β*_,*H*_*N*_,*H*_*α*_,*N*.

The covariance matrix Σ_v_ is 6 × 6dimension, quantifying correlations between the chemical shifts of six nuclei.

$$\sum_{v}=\left[\begin{array}{l} \sigma^{v}{}_{1,1}\;\sigma^{v}{}_{1,2}\;\cdots \;\sigma^{v}{}_{1,6}\\ \sigma^{v}{}_{2,1\;}\sigma^{v}{}_{2,2}\;\cdots \;\sigma^{v}{}_{2,6}\\ \;\vdots \;\vdots \;\vdots \\ \sigma^{v}{}_{6,1}\;\sigma^{v}{}_{6,2}\;\cdots \;\sigma^{v}{}_{6,6} \end{array}\right]$$

where the element

$$\sigma_{i,j}^{v}=\frac{1}{p_{v}}\sum \;\left(t_{i}-\mu_{i}^{\left(v\right)}\right)\;\left(t_{j}-\mu_{j}^{\left(v\right)}\right)$$ (10)

here *v* = beta-hairpin, not beta-hairpin; i,j = *C*,*C*_*α*_,*C*_*β*_,*H*_*N*_,*H*_*α*_,*N*

From Eq (6) and Eq (7), we have concluded

$$\xi_{ij}=\eta_{i}-\eta_{j}$$ (11)

It can be easily proved that *p*(*w*_*k*_ *|X*) is the maximum of *p*(*w*_*v*_ *|X*), if *η*_*k*_ is the maximal one in *η*_*v*_ (*v* = beta-hairpin, not beta-hairpin). Then, we predict that *X* belongs to group *k*. In statistical results, fluctuation phenomenon inevitably exists. To correct predicted results, we define the coefficient of the error allowed scope as

$$R=\frac{\eta_{corr}-\eta_{wro}}{\eta_{corr}}$$ (12)

where *η*_*corr*_ denotes *X* belonging to itself class *η*, *η*_*wro*_ denotes *X* being predicted other class *η*. Set the appropriate *R*, the sequence *X* in the error allowed scope can be classified correctly by using Eq (12).

### Performance evaluation

In statistical prediction, the jackknife test is considered to be the most rigorous test method [36] and has been widely used to evaluate the performance of various predictors [37–41]. However, considering the longer time needed for the jackknife test and because the goal of our paper concentrated on introducing a new model for beta-hairpin prediction, we adopted the three-fold cross-validation to evaluate the performance of our method. We randomly divided the training dataset into three parts, two of which are for training and the one for testing. The process is repeated three times. The final performance was calculated by averaging over all three datasets. The following parameters: the sensitivity (Sn), specificity (Sp), the overall accuracy (Acc) and Mathew’s correlation coefficient (MCC) are used to evaluate the predictive performance of our approach.

$$Sn=\frac{TP}{TP+FN}\times 100\%$$ (13)

$$Sp=\frac{TN}{TN+FP}\times 100\%$$ (14)

$$Acc=\frac{TP+TN}{TP+FN+TN+FP}\times 100\%$$ (15)

$$MCC=\frac{\left(TP\times TN)-(FP\times FN\right)}{\sqrt{\left(TP+FN)\times (TN+FN)\times (TP+FP)\times (TN+FP\right)}}\times 100\%$$ (16)

where true positive (*TP*) denotes the number of correctly predicted beta-hairpin motif, false negative (*FN*) denotes the number of the beta-hairpin misclassified as not beta-hairpin motif, false positive (*FP*) denotes the number of the not beta-hairpin misclassified as beta-hairpin motif, and true negative (*TN*) denotes the number of correctly predicted not beta-hairpin motif.

## Results and Discussion

### Statistical distribution of the average CSs of six nuclei

**Fig 1:**
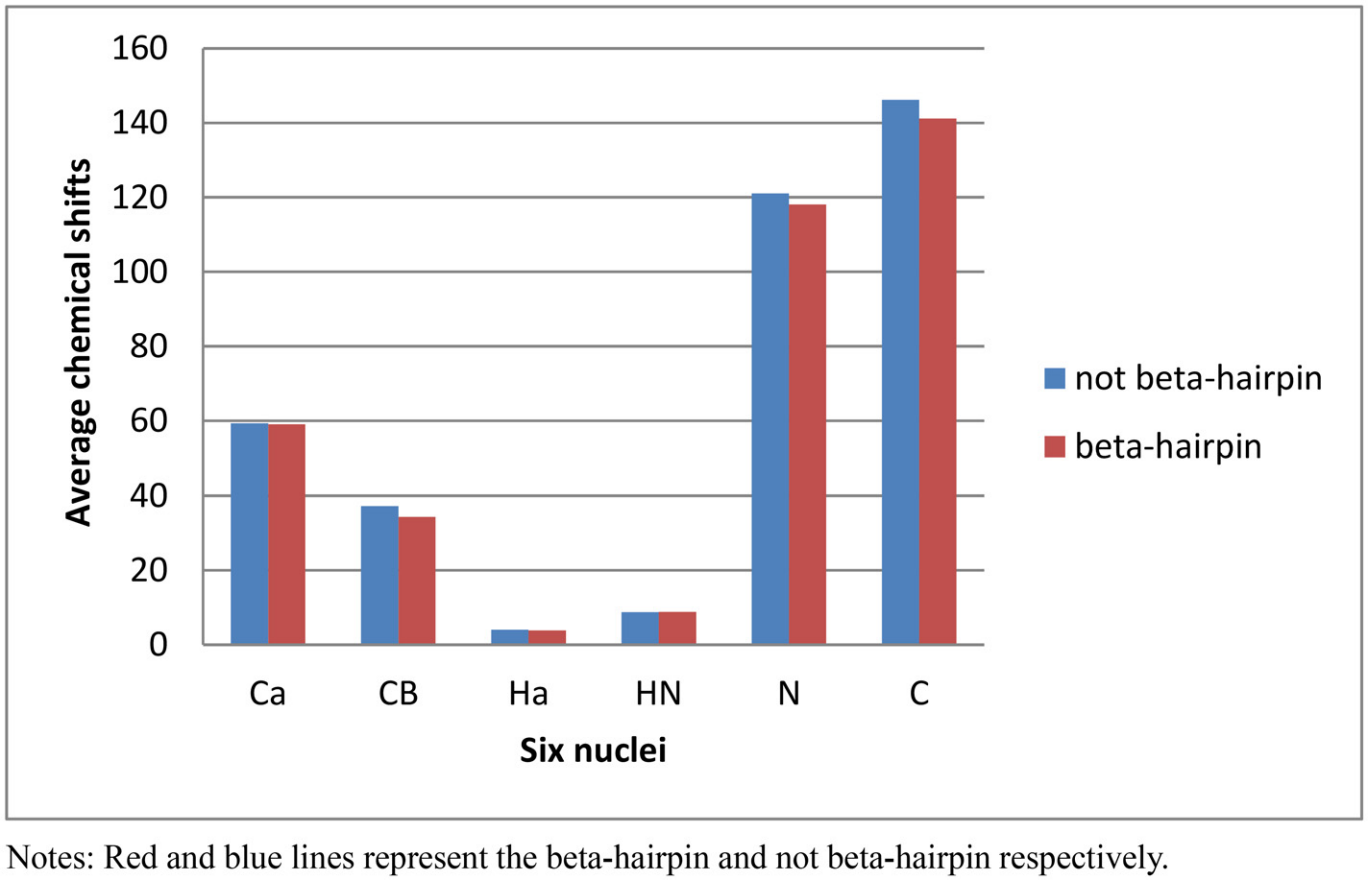
Distribution chart of six-nuclei CSs in beta-hairpin and not beta-hairpin motifs.

We analyzed the average chemical shifts of six nuclei in beta-hairpin and not beta-hairpin dataset. As showed in Fig 1, we found that the different distribution of the CSs six nuclei in beta-hairpin and not beta-hairpin dataset. The average chemical shift values of *C*,*C*_*α*_,*C*_*β*_,*H*_*α*_,*N* nuclei are higher in not beta-hairpin dataset than beta-hairpin dataset. However, the average chemical shift value of *H*_*N*_ nuclei is lower in not beta-hairpin dataset than beta-hairpin dataset.

For further investigating whether the distribution of average CSs of six nuclei in two datasets are independent of one another, the analysis of variance (ANOVA) [19, 31] can be used for the average CSs of six nuclei in beta-hairpin and not beta-hairpin statistical analysis under a normal distribution. Though we know that many test statistics are approximately normally distributed for large samples (generally>30 samples) under the central limit theorem. In order to strictly verify the validity of a normal distributional assumption, we implemented the statistical test. The Quantile-quantile (Q-Q) plot or Probability-probability (P-P) plot in statistics is often as a means to check the validity of a statistical distributional assumption for a dataset [42]. In term of P-P plot, if the data indeed follow the assumed normal distribution, then the points on the P-P plot will fall approximately on the diagonal line. The result demonstrated that the sampling distributions of six-nuclei CSs obey normal distribution (see supplementary material S4 file). Therefore, ANOVA can be implemented. Table 1 records the F-values of six nuclei and corresponding *p*-values. From Table 1 we observed that six *p*-values are less than 0.05 (*p*< 0.05). This result shows that the average CSs of six nuclei have a significant difference between beta-hairpin and not beta-hairpin structures, suggesting that beta-hairpin motifs can be discriminated from not beta-hairpin sequences based on the CSs of six nuclei.

**Table 1:** The statistical test using ANOVA for CSs of six nuclei.

Nuclei	ANOVA (*p*–value)
*C*	4.42 (*p*<0.05)
*C*_*a*_	4.01 (*p*<0.05)
*C*_*B*_	4.44 (*p*<0.05)
*H*_*N*_	4.13 (*p*<0.05)
*H*_*a*_	4.36 (*p*<0.05)
*N*	4.12 (*p*<0.05)

### Prediction of beta-hairpin based on the CSs of six nuclei

Results in Table 1 suggest that the CSs of six nuclei are capable of predicting beta-hairpin. Therefore, we examined the accuracy of six nuclei by using QDA algorithm. Under the benchmark dataset, we calculated the average chemical shift values using the Eq (1). The sequences from two data subsets are converted respectively into six-dimensional vectors. In the training sets, determinant and inverse matrix of covariance matrix Σ_*v*_ are calculated. And *μ* is a six-dimensional mean vector, which is calculated in each dataset. Given a sequence *X* in testing sets, we may calculate *η*_*v*_ by using Eqs (6–11) and compare the results. Then the class of sequence *X* was determined by the maximum of *η*_*v*_ (*v* = beta-hairpin and not beta-hairpin). Finally, the coefficient *R* given in Eq (12) is used to correct predicted results. The current study utilized *R*<0.2. The results of three-fold cross-validation are listed in Table 2.

**Table 2:** Results of different parameters using QDA (R<0.2).

Parameters	Sn	Sp	Acc	MCC
Six CSs	92%	94%	87%	0.85
20 AAC	36%	87%	32%	0.26

From the Table 2, we can see that the sensitivity, specificity and total accuracy are 92%, 94% and 87%, respectively, indicating that chemical shift is a good parameter for the beta-hairpin prediction.

Chemical shift is an easily obtained experimental datum. However, Chemical shift values of a sequence are not always complete for a multitude of reasons. Often, chemical shifts can only be assigned partially or are missing. To assess the impact of incomplete chemical shift assignment and determine the importance of chemical shift of each nucleus, we performed the prediction by removing any one of the CSs six nuclei. Then, the CSs of combination of five nuclei can be seen as features to predict the beta-hairpin. The results are listed in Table 3.

**Table 3:** Predicted results by using the CSs of five nuclei (R<0.2).

Parameters	Sn	Sp	Acc	MCC
*C*_*α*_,*C*_*β*_,*H*_*N*_,*H*_*α*_,*N*(omit *C*)	98%	52%	83%	0.61
*C*,*C*_*β*_,*H*_*N*_,*H*_*α*_,*N*(omit *C*_*a*_)	94%	48%	80%	0.50
*C*,*C*_*α*_,*H*_*N*_,*H*_*α*_,*N*(omit *C*_*B*_)	87%	76%	83%	0.62
*C*,*C*_*α*_,*C*_*β*_,*H*_*α*_,*N*(omit *H*_*N*_)	100%	48%	83%	0.61
*C*,*C*_*α*_,*C*_*β*_,*H*_*N*_,*N*(omit *H*_*a*_)	94%	32%	74%	0.35
*C*,*C*_*α*_,*C*_*β*_,*H*_*N*_,*H*_*α*_(omit *N*)	100%	14%	72%	0.29

In table 3, we can see that all results are affected compared with using six CSs as features when a CSs feature is left out. If all six CSs are used, we reach a prediction overall accuracy of 87% (see Table 2). The absence of one CS leads to a significant decrease in prediction accuracy ranging from 4% for missing *C* or *C*_*B*_ or *H*_*N*_ shifts to 15% for missing *N* shifts. It is strange that the overall accuracy is worst when the CS of *N* nuclei is left out. This illustrates that *N* is the most important feature for prediction the beta-hairpin. According to the overall accuracy, we rank as the importance as: *N*>*H*_*a*_>*C*_*a*_>*C*> *H*_*N*_ >*C*_*B*_ in this paper.

### Comparison with other feature

To test our method and facilitate comparison with other feature, we used 20 amino acid compositions (AAC) as inputs of the method of QDA. Notes: Where *μ* is a twenty-dimensional mean vector, and Σ_*v*_ denotes the 20×20 dimensional covariance matrix. The results are also recorded in Table 2. Compared results show that the performance of CSs is more superior to that of 20 AAC for the beta-hairpin prediction.

### Comparison with other approaches

Some approaches have been developed for predicting the beta-hairpin motifs [7–10]. However, due to differences in database, it is difficult to directly compare our results with other published results. Here we examined the predicted performance of other algorithms by use of the same CSs of six nuclei as features. At present, the support vector machine (SVM) and random forest (RF) are arguably the most widely used classification techniques in the Life Sciences [43–46]. In this paper, we implemented the SVM and RF algorithm based on R software package. The results are all listed in Table 4.

**Table 4:** The results of different approaches using the same six CSs information.

algorithm	Sn	Sp	Acc	MCC
QDA	**92%**	94%	**87%**	**0.85**
SVM	71%	**98%**	86%	0.75
RF	12%	**86%**	62%	0.28

Table 4 shows that QDA yields the best outcomes in using six CSs as feature. Therefore, we proposed using QDA to perform the beta-hairpin motifs prediction.

## Conclusion

In this paper, we have introduced a model for predicting beta-hairpin motifs based on CSs. By the analysis of the statistical distributions of six-nuclei CSs in beta-hairpin and not beta-hairpin dataset, we found that the CSs of six nuclei are significantly different in beta-hairpin and not beta-hairpin motifs. Finally, we adopted three-fold cross-validation, and achieved the best prediction, namely the sensitivity (Sn) of 92%, the specificity (Sp) of 94%, the total accuracy (Acc) of 87% with 0.85 of Mathew’s correlation coefficient (MCC) by using six CSs as features and the quadratic discriminant analysis. Results showed that chemical shift is indeed an effective parameter for the prediction of beta-hairpin motifs. Moreover, we have performed the prediction by combining the CSs of five different nuclei. Results showed that CSs of each nucleus has a different influence on the prediction of beta-hairpin structures. Our model is both simple and easy to perform. We hope this model will assist investigation the topology of protein structures in the near future [47–49]. As demonstrated in a series of recent publications [50–53] in developing new prediction methods, user-friendly and publicly accessible web-servers will significantly enhance their impacts [54], we shall make efforts in our future work to provide a web-server for the prediction method presented in this paper.

## Supporting Information

S1 File 123 proteins used in this paper. (DOCX)

S2 File CSs data of 157 beta-hairpin fragments. (RAR)

S3 File CSs data of 75 not beta-hairpin fragments. (RAR)

S4 File p-p plots of six nuclei. (DOC)

## Abbreviations

CSs: Chemical Shifts
Sn: Sensitivity
Sp: Specificity
MCC: Mathew’s correlation coefficient
Acc: The overall accuracy
AAC: Amino acid compositions
QDA: Quadratic discriminant analysis
ANOVA: Analysis of variance
