# Supplementary material for: Identify Beta-Hairpin Motifs with Quadratic Discriminant Algorithm Based on the Chemical Shifts
Source: PLoS One. 2015 Sep 30;10(9):e0139280. doi: 10.1371/journal.pone.0139280 (PMC4589334; doi:10.1371/journal.pone.0139280)
Supplement: S1 File — (DOCX) [file pone.0139280.s001.docx]

**123 proteins used in this work**

| 1a6j | 1g6h | 1r5r | 1iwm | 1h4a | 1dtl | 1enf | 1pht |
| --- | --- | --- | --- | --- | --- | --- | --- |
| 1ail | 1gnu | 1uoh | 1j54 | 1iko | 1fdq | 1ub4 | 1mr3 |
| 1akh | 1hh8 | 1byf | 1jrl | 1umu | 1fil | 1ek8 | 1m15 |
| 1am7 | 1iw0 | 1e0c | 1m1f | 1ejf | 1fzy | 1naq | 1gns |
| 1b2v | 1j1v | 1gxq | 1ng2 | 1ekg | 1g6a | 1uv0 | 1f2f |
| 1bed | 1j97 | 1h70 | 1qav | 1ew4 | 1iho | 1mxe | 1mjc |
| 1bgf | 1jr2 | 1jn3 | 1qfj | 1jhf | 1k6m | 1joc | 1n0s |
| 1bja | 1k82 | 1l6x | 1qg7 | 1jiw | 1php | 1hcb | 1hvc |
| 1b88 | 1lfo | 1o13 | 1vjh | 1osp | 1qqy | 1cyn | 1edh |
| 1c44 | 1lm4 | 1rsy | 1ycq | 1qj8 | 1rro | 1hrh | 1iaz |
| 1cex | 1nd4 | 1slm | 1bdo | 1qmy | 1rwy | 1a7g | 1gaw |
| 1cy5 | 1pz4 | 1tn3 | 1crb | 1uuh | 1tph | 1mmq | 1mo1 |
| 1dhn | 1q4r | 1f2l | 1dfu | 1ass | 1vap | 1jl3 | 1lw6 |
| 1dqe | 1qkr | 1f35 | 1epf | 1avs | 1by9 | 1huu | 1j7d |
| 1f3v | 1qst | 1go4 | 1gwy | 1col | 1dyt | 1ff3 | 1hsl |
| 1l1d | 1l0s | 1hfc |  |  |  |  |  |
