# Supplementary figures and images for: Identify Beta-Hairpin Motifs with Quadratic Discriminant Algorithm Based on the Chemical Shifts
Source: PLoS One. 2015 Sep 30;10(9):e0139280. doi: 10.1371/journal.pone.0139280 (PMC4589334; doi:10.1371/journal.pone.0139280)

P-P plots of six nuclei


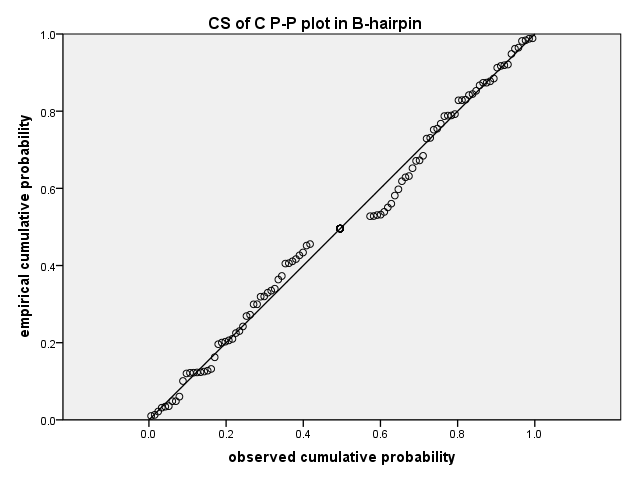


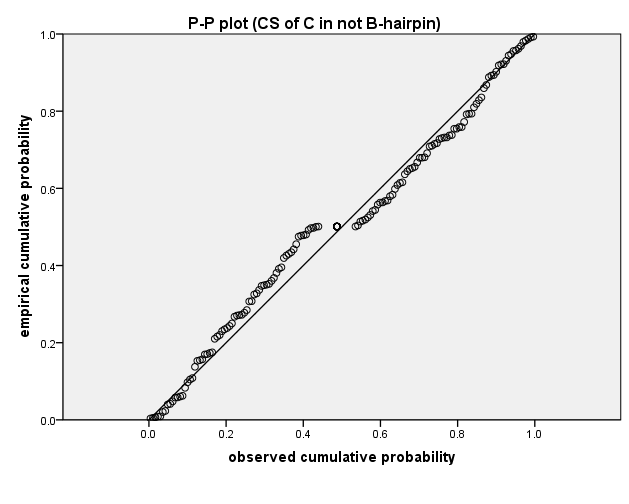


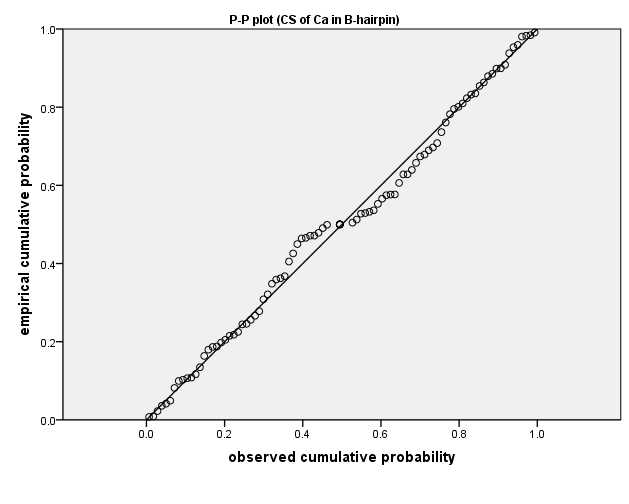


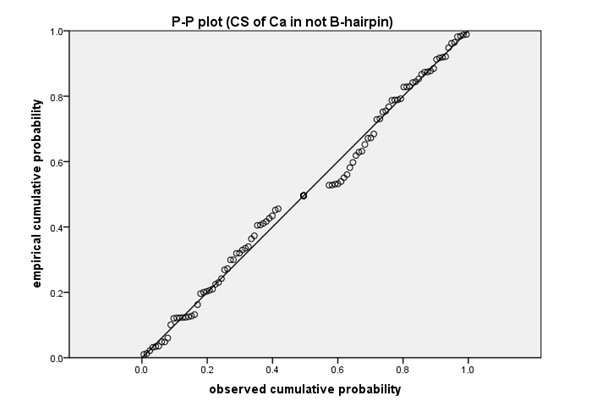


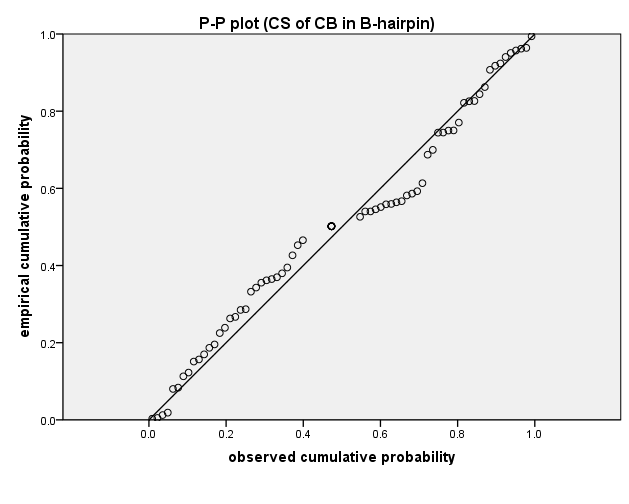


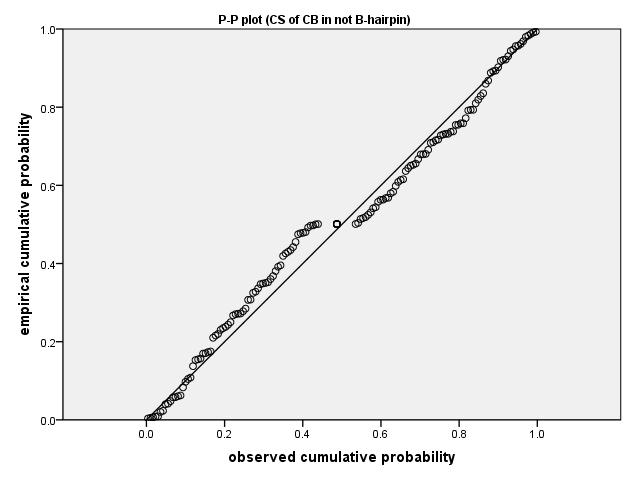


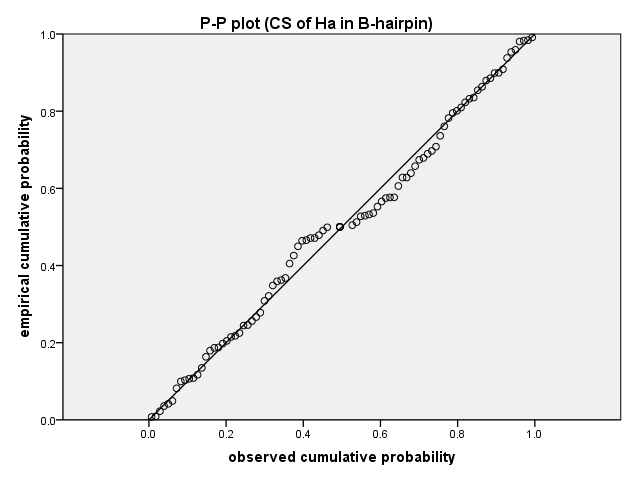


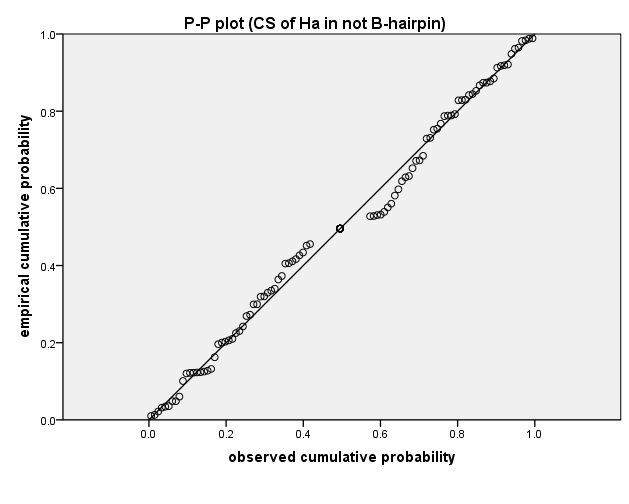


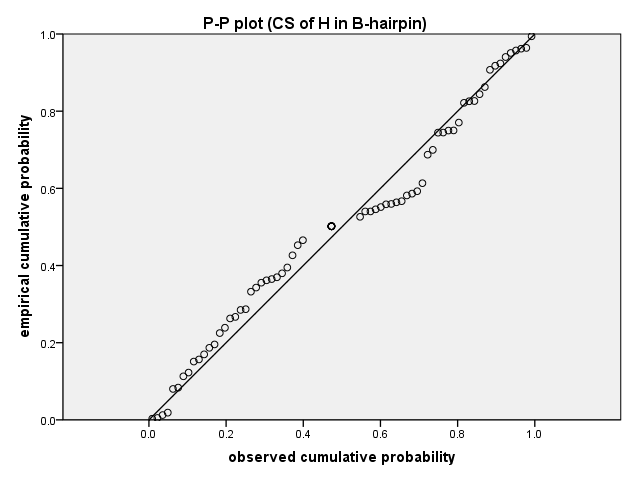


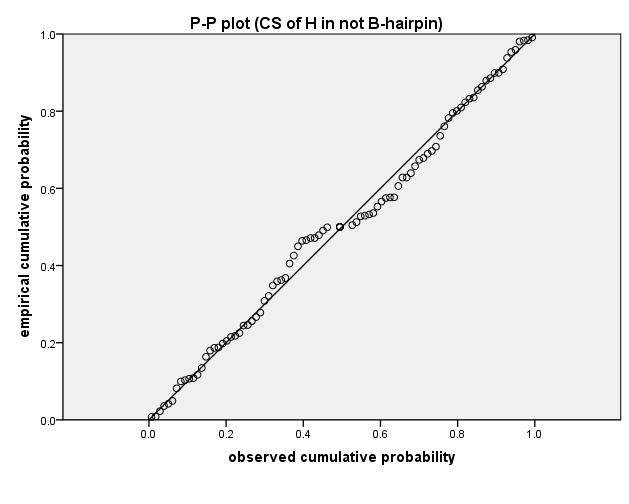


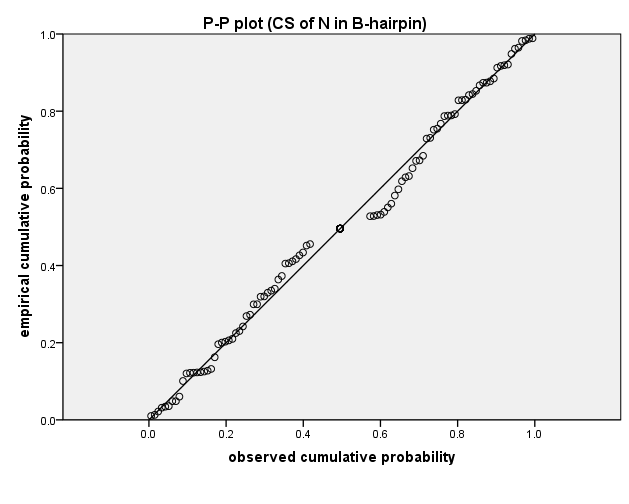


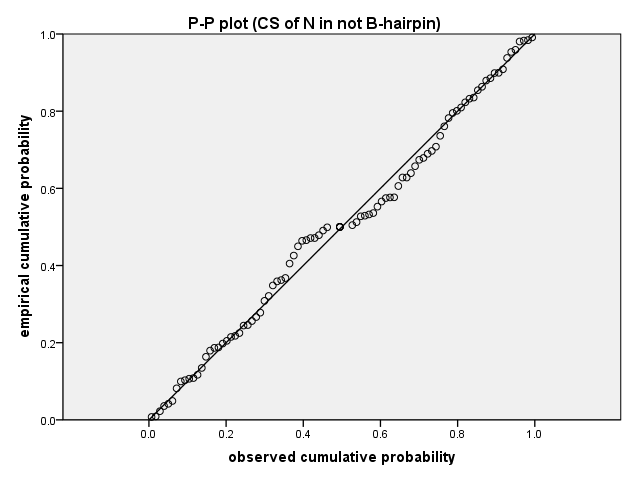

Supplement: S4 File — (DOC) [file pone.0139280.s004.doc]
